# Improved persistence and adherence to diuretic fixed-dose combination therapy compared to diuretic monotherapy

**DOI:** 10.1186/1471-2296-9-61

**Published:** 2008-11-06

**Authors:** Bimal V Patel, Rosemay A Remigio-Baker, Patrick Thiebaud, Ronald Preblick, Craig Plauschinat

**Affiliations:** 1 MedImpact Healthcare Systems, Inc., 10680 Treena Street, 5th Floor, San Diego, CA 92131, USA; 2 Evidence Based Medicine, Novartis Pharmaceuticals Corporation, 59 Route 10, East Hanover, NJ 07936, USA

## Abstract

**Background:**

Diuretics are recommended as initial treatment for hypertension. Several studies have suggested suboptimal persistence and adherence to thiazide diuretic monotherapy; this study compared patient persistence and adherence with hydrochlorothiazide (HCTZ) monotherapy to fixed-dose combinations containing HCTZ.

**Methods:**

Patients with at least one prescription claim during 2001 to 2003 for either HCTZ or one of the following fixed-dose combinations: angiotensin-receptor blockers/HCTZ (ARB/HCTZ), angiotensin-converting enzyme inhibitor/HCTZ (ACEI/HCTZ), or beta blockers/HCTZ (BB/HCTZ) were identified. Patients were required to be continuously benefit-eligible six months pre- and one year post-index date, and to have no prescription claims for any antihypertensive therapy six months prior to the index date. Patients were followed for one year to assess persistence, medication possession ratio (MPR), adherence (MPR >80%), and proportion of days covered (PDC) with initial antihypertensive therapy. Logistic regression was used to calculate adjusted odds ratios for persistence, adherence and PDC, adjusted for age, gender, business segment, RxRisk disease categories, average co-pay and concurrent cardiovascular-related medication utilization.

**Results:**

The study cohort consisted of 48,212 patients; 72.5% used HCTZ, 13.2% ACEI/HCTZ, 9.3% ARB/HCTZ, and 5.0% BB/HCTZ. Mean age was 53.7 years and 66.5% were female. A significantly lower proportion of patients using HCTZ (29.9%) remained persistent with therapy at 12 months compared with ARB/HCTZ (52.6%; OR = 0.37, CI = 0.36, 0.38), ACEI/HCTZ (51.4%; OR = 0.38, CI = 0.37, 0.39), and BB/HCTZ (51.9%; OR = 0.38, 0.37, 0.40). Similarly, PDC was lower for HCTZ patients (32.5%) as compared to ARB/HCTZ (53.7%; OR = 0.39, CI = 0.37, 0.40), ACEI/HCTZ (50.9%; OR = 0.42, CI = 0.40, 0.43), and BB/HCTZ (51.3%; OR = 0.44, CI 0.42, 0.45). MPR was also significantly lower for HCTZ patients as compared to those using fixed-dose combination therapies.

**Conclusion:**

Initiating HCTZ fixed-dose combination therapy with an ACEI, ARB, or BB was associated with greater persistence and adherence as compared to HCTZ monotherapy. Further research is needed to determine the relationship between improved persistence and adherence with blood pressure control.

## Background

Hypertension affects almost one in three American adults; age-adjusted prevalence in 2005 was estimated at 33.6% [1,2]. Only 37% of patients with hypertension, and only slightly more than half (57%) of those receiving antihypertensive treatment currently have their blood pressure (BP) controlled [1]. The Joint National Committee on Prevention, Detection, Evaluation, and Treatment of High Blood Pressure published its seventh report (JNC 7) in 2003 [3]. JNC 7 included the recommendation of thiazide diuretics, either alone or in combination with drugs from other classes, as initial therapy treatment of most patients with hypertension.

Patient persistence and adherence to prescribed antihypertensive therapy is a key component of hypertension management. Greater persistence with antihypertensive therapy has been associated with lower rates of long-term hypertension sequelae [4], as well lower health care resource use [5-8] and hospitalization rates [6]. In usual-practice settings, less than optimal persistence with antihypertensive monotherapy regimens has been well documented [9-13]. Several studies have indicated that initial monotherapy treatment with diuretics is associated with poorer patient persistence, compared to angiotensin-converting enzyme inhibitors (ACEI), angiotensin-receptor blockers (ARB), and beta blockers (BB). [9,11,13]. Tolerability and perceived side effects associated with antihypertensive medications may play an important role in patient motivation, and thus affect medication persistence [14]. Patients may complain of frequent urination upon initiation of diuretic therapy, and diuretics have been associated with side effects such as weakness, fatigue, palpitations, and electrolyte disturbances. Patients are often prescribed a lower dose of diuretic when used in combination therapy with an agent from another antihypertensive class, and combination low-dose therapy has been shown to increase BP-lowering efficacy and reduce adverse side effects associated with higher-dose monotherapy regimens [15]. Thus, the addition of a medication from another antihypertensive class to a diuretic may attenuate the side effects often seen with diuretics when used as monotherapy [15-17].

The purpose of this study was to compare patient persistence and adherence to hydrochlorothiazide (HCTZ) monotherapy versus fixed-dose combinations containing HCTZ and an ACEI, ARB, or BB in a natural (non-clinical trial) setting. This retrospective, longitudinal cohort study employed administrative pharmacy claims data to examine drug utilization in patients previously naïve to antihypertensive therapy who initiated therapy with HCTZ monotherapy, or fixed-dose ACEI/HCTZ, ARB/HCTZ, or BB/HCTZ.

## Methods

This was a retrospective, population-based study which employed a pharmacy claims database from MedImpact, a large US pharmacy benefits manager (PBM) which administers prescription benefits for about 27 million persons across the US. Adult participants (≥ 18 years) were eligible for study inclusion if they received ≥ 1 prescription for HCTZ or fixed-dose combination ACEI/HCTZ, ARB/HCTZ, or BB/HCTZ during the study identification period of January 1, 2001 through December 31, 2003. "Index drug" was defined as the first prescription therapy filled within the identification period, and the "index date" was defined as the date of the first index drug fill. Participants were required to be continuously benefit-eligible for at least 6 months preceding and 12 months subsequent to the index date. Patients were required to have no claims for any antihypertensive therapy during the 6 months prior to their index date.

**Figure 1:**
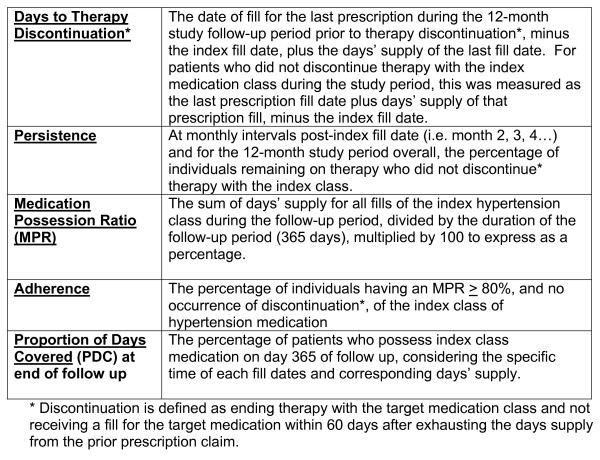
Study utilization metrics and definitions.

Drug utilization was followed for 1 year subsequent to the index date, and analyses were performed relative to the specific index drug classes. Figure 1 includes definitions of utilization metrics, including persistence, medication possession ratio (MPR), adherence, proportion of days covered (PDC) and time to therapy discontinuation. For index drug classification purposes, patients who received prescription fills for antihypertensive medications in addition to the study index medication on their study index date were excluded.

RxRisk [18] methodology was used to identify the presence of other diseases. RxRisk identifies claims during the 6 months prior to the index date for medications used to treat specific conditions to identify co-morbidities (e.g. a pharmacy claim for an oral antidiabetic agent is used to identify the presence of diabetes). RxRisk disease categories were used (e.g. diabetes yes/no) to describe patient clinical characteristics, and for adjustment of statistical comparisons for the presence of co-morbid conditions.

To control for potential physician's treatment selection bias, a propensity score adjustment was performed. A multinomial regression model was constructed to estimate the probability of the observed treatment choice based on patient characteristics, including patient age, gender, RxRisk disease categories, and type of benefit coverage (i.e., Commercial, Medicaid, Medicare, and self-insured). The inverse of this probability, or propensity score weight, was then used in study multivariable logistic and linear regressions. For MPR, multiple variable linear regression techniques were used in pairwise comparisons of means adjusted for independent variables, which included propensity weight, patient age, gender, RxRisk disease categories, type of benefit coverage, average co-pay, and concurrent cardiovascular-related medications utilized (i.e., other antihypertensive drugs not equal to the initial therapy subsequent to the index pharmacy claim date; and digitalis, nitrates, antiplatelet agents and antihyperlipidemics). Pairwise multiple logistic regression was used to calculate odds ratios to compare persistence, adherence, and proportion of days covered (PDC), adjusted for the same independent variables, using HCTZ monotherapy as the reference group. Although these statistical methods were used to control for important variables, crude statistics are included in the text and the tables for descriptive purposes, since statistical adjustment did not appreciably alter any values.

Persistence was evaluated using multivariable survival analysis techniques. Cox proportional hazards regression was employed to calculate hazard ratios (HR) to compare discontinuation for antihypertensive medications studied, adjusted for covariates. Discontinuation of therapy with the index medication was modeled using HCTZ as the referent. All analyses for this study were performed using SAS version 9.1.3 (SAS Institute, Inc, Cary, NC).

## Results

The final study population was comprised of 48,212 patients newly initiated on antihypertensive therapy. Cohort descriptive characteristics are summarized in Table 1. Overall, 67% were females and the mean age was 53.7 years. HCTZ users comprised 72.5% of the study cohort, while 13.2% used ACEI/HCTZ, 9.3% ARB/HCTZ, and 5.0% BB/HCTZ. HCTZ and BB/HCTZ patients were generally younger than ACEI/HCTZ or ARB/HCTZ patients. Patients receiving HCTZ were more likely to be female than those utilizing fixed-dose combination therapy. The proportion of cohort patients with cardiovascular-related conditions or diabetes was higher for ACEI/HCTZ and ARB/HCTZ than for HCTZ or BB/HCTZ.

**Table 1:** Study cohort descriptive characteristics (N = 48,212)

	Initial anti-hypertensive therapy			
Characteristics	HCTZ (N = 34,934, 72.5%)	ARB/HCTZ (N = 4,469, 9.3%)	ACEI/HCTZ (N = 6,388, 13.2%)	BB/HCTZ (N = 2,421, 5.0%)
Mean age in years ± SD	53.6 ± 15.3	54.6 ± 13.8	54.3 ± 13.9	52.2 ± 13.8
Age in categories				
18 – 44 (%)	10,296 (29.5)	1,006 (22.5)	1,486 (23.3)	712 (29.4)
45 – 54 (%)	9,769 (28.0)	1,457 (32.6)	2,021 (31.6)	780 (32.2)
55 – 64 (%)	6,423 (18.4)	1,006 (22.5)	1,469 (23.0)	505 (20.9)
65 – 74 (%)	4,500 (12.9)	570 (12.8)	824 (12.9)	256 (10.6)
75 – 84 (%)	2,925 (8.4)	327 (7.3)	443 (6.9)	115 (4.8)
85 and above (%)	1,021 (2.9)	103 (2.3)	145 (2.3)	53 (2.2)
Frequency female (%)	24,495 (70.1)	2,573 (57.6)	3,629 (56.8)	1,356 (56.0)
RxRisk^a, b^				
Behavioral Health	6,413 (18.4)	653 (14.6)	810 (12.7)	332 (13.7)
Cardiovascular Conditions	2,012 (5.8)	435 (9.7)	874 (13.7)	128 (5.3)
Gastric acid disorder, IBS	2,768 (7.9)	250 (5.6)	351 (5.5)	147 (6.1)
Asthma, Allergic Rhinitis	2,590 (7.4)	196 (4.4)	301 (4.7)	79 (3.3)
Diabetes	843 (2.4)	147 (3.3)	281 (4.4)	23 (1.0)
Thyroid Disease	2,193 (6.3)	230 (5.1)	321 (5.0)	108 (4.5)
Rheumatoid Arthritis, Gout	1,273 (3.6)	120 (2.7)	171 (2.7)	50 (2.1)
Primary market segment				
HMO	24,562 (70.3)	2,644 (59.2)	4,656 (72.9)	1,631 (67.4)
Medicaid	4,103 (11.8)	125 (2.8)	300 (4.7)	112 (4.6)
Medicare	2,285 (6.5)	227 (5.1)	355 (5.6)	122 (5.0)
Self-insured	3,984 (11.4)	1,473 (33.0)	1,077 (16.9)	556 (23.0)
Average copay ($)^c^	5.2 ± 4.2	22.8 ± 19.0	14.0 ± 13.0	9.2 ± 8.7

^a ^The 49 RxRisk categories were consolidated into 21 categories of which the top 7 most prevalent groups (3% threshold) are presented here. Behavioral health = anxiety and tension, bipolar, depression, psychotic illness, attention deficit disorder; Cardiovascular conditions: Cardiac disease, coronary & peripheral vascular disease, heart disease, hypertension (does not include the target medications, digitalis, nitrates, anti-platelet agents or anti-hyperlipemic agents); Gastrointestinal (GI) disorders = Gastric acid disorder, irritable bowel syndrome

^b ^Limited to antihypertensive medications of the same class as the index drug

Unadjusted results for antihypertensive medication utilization metrics are displayed in Table 2. Patients receiving HCTZ had the lowest persistence (29.9%), compared to 52.6% for ARB/HCTZ, 51.9% for BB/HCTZ, and 51.4% for ACEI/HCTZ. The MPR was highest for BB/HCTZ (62.1%), followed by ARB/HCTZ (60.5%), ACEI/HCTZ (58.3%), and HCTZ (44.5%). Adherence was lowest for HCTZ patients (24.2%) and highest for BB/HCTZ (43.9%). HCTZ patients had the lowest proportion of days covered (32.5%), and time to therapy discontinuation was longest for ARB/HCTZ patients (240.1 ± 140.3 days).

**Table 2:** Unadjusted antihypertensive persistence and adherence

Outcome measures	HCTZ	ARB/HCTZ	ACEI/HCTZ	BB/HCTZ
Persistence (%)	29.9	52.6	51.4	51.9
Adherence (%)	24.2	39.2	38.8	43.9
PDC^a ^at end of follow-up (%)	32.5	53.7	50.9	51.3
MPR^b ^± SD	44.5 ± 34.5	60.5 ± 32.7	58.3 ± 34.2	62.1 ± 34.1
Days to therapy discontinuation ± SD	164.5 ± 141.8	240.1 ± 140.3	235.9 ± 140.8	238.2 ± 140.9

^a^PDC denotes proportion of days covered at the end of the follow-up period. The percentage of patients who possess index class medication on day 365 of follow-up period.

^b^MPR denotes medication possession ratio.

Adjusted odds ratios for persistence, adherence, and days covered are found in Table 3. Compared to all fixed-dose combination therapy users, HCTZ patients were significantly (p < 0.01, all comparisons) less likely to be persistent, adherent, and to have medication "on hand" at the end of the study period. Patients receiving HCTZ were approximately 63% less likely to be persistent compared to ARB/HCTZ patients, 62% less likely to be persistent than ACEI/HCTZ patients, and 61% less likely to be persistent than BB/HCTZ patients. Similarly, HCTZ patients were 60%, 54%, and 50% less likely to be adherent as compared to BB/HCTZ, ARB/HCTZ, and ACEI/HCTZ patients, respectively. Odds ratios for days' covered indicated that HCTZ users were 61%, 59%, and 56% less likely to have medication on hand at the end of the study as compared to ARB/HCTZ, ACEI/HCTZ, and BB/HCTZ patients, respectively. Comparisons for MPR (Table 3) also revealed that HCTZ patients had lower adjusted mean MPR as compared to BB/HCTZ (difference = -17.0, p < 0.0001), ARB/HCTZ (difference = -16.6, p < 0.0001), and ACEI/HCTZ (difference = -12.9, p < 0.0001).

**Table 3:** Outcome measures – adjusted pairwise comparison of HCTZ monotherapy versus each fixed-dose combination therapy

	HCTZ fixed-dose combination therapy		
	
Outcome measures	ARB/HCTZ	ACEI/HCTZ	BB/HCTZ
Persistence (Odds Ratio, 95% CI)	0.369^a^ (0.356, 0.383)	0.380^a^ (0.368, 0.393)	0.382^a^ (0.370, 0.395)
Adherence (Odds Ratio, 95% CI)	0.457^a^ (0.440, 0.475)	0.495^a^ (0.478, 0.513)	0.398^a^ (0.385, 0.412)
PDC at end of follow-up period (Odds Ratio, 95% CI)	0.388^a^ (0.374, 0.403)	0.415^a^ (0.402, 0.429)	0.435^a^ (0.421, 0.449)
MPR (HCTZ vs. fixed-dose)	44.4 vs 61.0 Δ = -16.6^b^	44.6 vs 57.5 Δ = -12.9^b^	44.5 vs 61.5 Δ = -17.0^b^

^a ^Significant difference in outcome measures between pairwise comparison with HCTZ users at p < 0.05.

^b ^p < 0.0001

Cox proportional hazards model results are included in Table 4. Patients receiving HCTZ were more likely to discontinue the index medication class as compared to users of ACEI/HCTZ, ARB/HCTZ, and BB/HCTZ, adjusted for demographic and clinical covariates. Cardiovascular-related conditions and patient copay were not significant predictors of therapy discontinuation in the proportional hazards model. An increased hazard of therapy discontinuation was observed for Medicaid and Medicare patients as compared to managed care patients, and for patients with diabetes.

**Table 4:** Results of Cox proportional hazards model for therapy discontinuation

	Study population (N = 48,212)	
Variable	Hazards ratio	p-value
Age	0.989	<.0001*
Female	1.048	0.0002*
RxRisk categories^a^		
Anxiety and Tension, Bipolar Disorder, Depression, Psychotic Illness, ADD	1.079	<.0001*
Asthma, Allergic Rhinitis	1.031	0.1891
Cardiac Disease, Coronary & Peripheral Vascular Disease, Heart Disease, Hypertension^b^	0.973	0.2432
Gastric acid disorder, IBS	1.004	0.8477
Rheumatoid Arthritis, Gout	1.147	<.0001*
Thyroid Disease	0.951	0.0426*
Diabetes	1.158	<.0001*
Business Type		
HMO	N/A	N/A
Medicaid	1.339	<.0001*
Medicare	1.451	<.0001*
Self	1.056	0.0013*
Concomitant Other CVD-related Medications Used		
Digitalis	1.138	0.0086*
Nitrates	1.224	<.0001*
Antiplatelet Medications	1.097	0.0502
Antihyperlipidemic Medications	0.797	<.0001*
Average copay^c^	1.001	0.3831
Target medication classes^d^		
ARB/HCTZ	0.529	<.0001*
ACEI/HCTZ	0.536	<.0001*
BB/HCTZ	0.532	<.0001*

* Significant p-value

^a ^RxRisk categories shown in this table include the 7 most frequent disease states in the study cohort. All RxRisk disease categories were included in the model.

^b ^This RxRisk category does not include the target medications, digitalis, nitrates, antiplatelet medications or antihyperlipidemics.

^c ^Limited to HTN medications of the same class as the index drug.

^d ^Reference Medication Class = HCTZ

## Discussion

Our retrospective study found that the combination of another antihypertensive medication with HCTZ via fixed-dose combination therapy was associated with better patient persistence and adherence as compared to HCTZ monotherapy. HCTZ patients were 61–63% less likely to be persistent, and 50–60% less likely to be adherent, than patients who initiated antihypertensive therapy with fixed-dose ACEI/HCTZ, ARB/HCTZ, or BB/HCTZ therapy. Patients were more likely to stay on fixed-dose combinations longer than monotherapy, as mean time to therapy discontinuation was, on average, two and a half months longer for fixed-dose combinations than for monotherapy. To our knowledge, the current study is the first to compare persistence with HCTZ monotherapy to fixed-dose HCTZ combination therapy in a "usual care" setting in the US.

A few other studies have used retrospective methods to evaluate combination therapy in "usual-care" settings outside of the US. Van Wijk et al, in a recent community-based retrospective study using pharmacy dispensing records in the Netherlands, studied 2325 previously naïve patients who newly initiated antihypertensive therapy with a ACEI, BB, calcium channel blocker (CCB), or diuretic [19]. Only 39% of patients used antihypertensive therapy consistently during 10 years of follow-up. More patients who initiated therapy with diuretics and BB discontinued compared to those who started with a CCB or ACEI. In this study, comparing patients who initiated with diuretics, those who started with fixed-dose combination therapy were 70% more likely to be persistent with therapy (OR 0.29; 95% CI 0.14–0.54).

Another recent study comparing utilization for antihypertensive monotherapy versus fixed-dose combination therapy used a Canadian database, and found that during the first 6 months after treatment was initiated, persistence declined to 75%; at the end of 3 years, only 55% were persistent [20]. During the first year of follow-up, compared to diuretic monotherapy, patients prescribed other antihypertensive classes or fixed-dose combination therapy (HR 0.71; 95% CI 0.67 to 0.75) were found to have higher persistence [20]. Other studies comparing monotherapy regimens conducted in naturalistic settings have consistently found poorer compliance and/or persistence for diuretics as compared to other antihypertensive therapeutic classes [11-13,21,22]. Conlin and colleagues [9] followed patients from a large pharmacy benefits manager (PBM) and demonstrated that at 4 years post-therapy initiation, only 16% of diuretic patients were persistent, compared to 61% of ARB, 47% of ACEI, 41% of CCB, and 35% of BB patients.

Improving patient adherence and persistence with antihypertensive therapy may have a beneficial effect on blood pressure control. One recent retrospective study of 840 patients using antihypertensive monotherapy assessed the relationship between medication compliance and blood pressure control (<140/90 mmHg or < 130/85 mmHg for diabetic hypertensive patients) [23]. Patients received monotherapy with an ACEI (27%), or CCB (22%), BB (20%), or diuretic (11%) and were classified as having high (80–100%), medium (50–79%), or low (<50%) medication compliance. High-compliance patients were 45% more likely to achieve blood pressure control than those with medium or low compliance after controlling for age, gender, and comorbidities.

Differences between antihypertensive drug regimens for patient medication adherence and persistence may have cost implications. Increased healthcare expenditures for nonadherent patients with hypertension in usual-care settings have been well documented [5-8]. In one study, Medicaid patients with an interruption of antihypertensive therapy consumed extra healthcare costs of $873/patient during the first year [5]. Another study of both MCO and traditional fee-for-service patients from a large PBM found that better compliance with antihypertensive therapy was associated with a decreased risk of hospitalization and, thus, lower medical costs [6]. Addressing patient noncompliance with antihypertensive medication can thus play an important role in managing the costs of patient care for MCOs and other healthcare providers.

Retrospective analyses of administrative databases can provide information about patient behavior in a naturalistic setting that is difficult to assess in clinical trials, but it is important to note some study limitations. Since definitions of adherence and persistence may be expected to differ somewhat between studies, comparisons of medication utilization results across studies should be interpreted cautiously. As medical claims were unavailable to confirm hypertension diagnosis, some of the patients included may not have had a diagnosis of hypertension, and may have been prescribed a study medication for an unrelated diagnosis. This may have particularly influenced patient selection for BB/HCTZ users, as this combination has multiple indications and may be used more frequently in patients with stable angina or acute coronary syndromes. Drug utilization metrics were measured by prescription refill patterns and not actual drug taken by the patient; however, other studies have supported the evaluation of pharmacy claims data for such purposes [24,25]. Propensity score weighting was used in statistical analyses to control for selection bias, but it is possible that factors that were not available for analysis may have played a role in physician treatment selection. Since patient BP was not available for analysis, patients prescribed HCTZ in combination with a drug from another antihypertensive class may have had a higher baseline BP than those prescribed HCTZ alone; this information was unavailable for inclusion in the propensity score adjustment and may have influenced our study's results. It could be hypothesized that patients prescribed HCTZ alone may be "healthier" patients than those prescribed HCTZ in combination. While analysis of the prevalence of comorbid conditions between treatment groups suggests that this may be somewhat true for HCTZ monotherapy patients as compared to ARB/HCTZ and ACEI/HCTZ patients, frequency of cardiovascular diagnoses and diabetes were similar for HCTZ and BB/HCTZ patients, yet persistence and adherence for BB/HCTZ patients was much greater than for HCTZ patients. Statistical analyses were employed to control for such differences in patient co-morbidity and demographic characteristics, and significant differences in therapy discontinuation rates persisted. Since we identified patients using a 6-month prior eligibility criteria, it is possible that some patients may have discontinued previous AHY therapy prior to the 6 months or received another AHY therapy while a member of another health plan. It is possible that a patient who discontinued a study medication did so based on physician instructions, and this would have been incorrectly classified as non-persistence and may have influenced study results. Finally, our study included only fixed-dose combinations with HCTZ and excluded free combination regimens. Other studies have demonstrated improved persistence with fixed-dose antihypertensive combination regimens as compared to free combinations [26]; therefore, some of the persistence advantages found in our study of fixed-dose combinations may not be evident when using free combination regimens.

This study sought to evaluate patient utilization of HCTZ monotherapy as compared to HCTZ used in combination in patients new to antihypertensive therapy. Our findings are not meant to imply that all patients prescribed HCTZ as monotherapy are candidates for initial combination antihypertensive therapy; important prescriber and patient clinical factors play a role in the choice of initial antihypertensive therapy. However, our study provides additional information regarding the use of these regimens in a non-clinical trial setting, and when interpreted in the context of other literature suggesting poor patient adherence and persistence with HCTZ monotherapy versus monotherapy with other antihypertensive classes, provides important information to prescribers of antihypertensive medication.

## Conclusion

In a naturalistic, non-clinical trial setting, our study suggested that patients who initiated new antihypertensive therapy with HCTZ in a fixed-dose combination with an ACEI, ARB, or BB may be more persistent than patients who initiated antihypertensive therapy with HCTZ alone. HCTZ patients were also less likely to be adherent as compared to fixed-dose combination patients, and to stay on therapy an average of 2.5 months less during the first year than patients using fixed-dose combinations. While additional research is needed to evaluate the impact of improved persistence and adherence with blood pressure control across practice settings, improving patient adherence and persistence with antihypertensive medication is an important approach to improving blood pressure control in the US.

## Competing interests

Dr. Plauschinat is employed by Novartis Pharmaceuticals Corporation, the manufacturer of several medications indicated for the treatment of hypertension, and also owns stock in Novartis. At the time of the study, Dr. Preblick was employed by Novartis Pharmaceuticals Corp., and Dr. Preblick owns Novartis stock. Dr. Patel, Ms. Remigio-Baker, and Dr. Thiebaud have no competing interests to disclose.

## Authors' contributions

BP and RP participated in conception and design of the study. BP, CP, RP, and RRB participated in writing and editing portions of the manuscript. RRB and PT participated in statistical analysis. All authors read and approved the final manuscript.

## Pre-publication history

The pre-publication history for this paper can be accessed here:


